# Exploring the Interplay Between Soil and Plants Under Biochar Application to Enhance Plant Resilience in a Changing Environment

**DOI:** 10.3390/plants14142181

**Published:** 2025-07-14

**Authors:** Genxing Pan, Stephen Joseph, Hans Peter Schmidt

**Affiliations:** 1 Institute of Resource, Ecosystem and Environment of Agriculture, and Center of Biochar and Green Agriculture, Nanjing Agricultural University, Nanjing 210095, China; 2 School of Environment and Natural Resources, Zhejiang University of Science and Technology, Hangzhou 310023, China; 3 School of Material Science and Engineering, University of New South Wales, Sydney, NSW 2052, Australia; 4 Ithaka Institute for Carbon Strategies, CH-1974 Arbaz, Switzerland

Plants are at the core of agriculture and human nutrition. Hence, plant health plays a pivotal role in human well-being. In this respect, the relation of soil and plant health should be highlighted to ensure food security and achieve the UN SDGs, encapsulated by the ONE Health approach [1,2]. However, the adequate functioning of soil fertility relies on soil organic matter, which has declined significantly due to intensive crop cultivation and tillage under warming and drier climate conditions. The application of biochar, thermo-converted from plant biomass, can be effective in improving soil quality and plant health, primarily by enhancing organic matter in soil [1,3].

Biochar has gained increasing prominence as a carbon-rich material obtained via plant biomass pyrolysis. It is used to amend soils, increase fertilizer efficiencies, remediate polluted land, and restore ecosystem functions [3,4,5,6]. Over the last decade, biochar’s role in promoting plant growth has been attributed to its improvement of the soil’s biophysical structure, nitrogen retention, and phosphorus and potassium availability [7], as well as toxicine immobilization [8,9]. Additionally, biochar may induce plant resistance through the soil biome [10]. Studies on soil–plant systems both in greenhouses and in the field [4,11,12,13] have provided evidence of increased resilience in biochar-induced systems against plant biotic stress from re-planting and pathogenic diseases, and abiotic stress from prolonged drought. Meanwhile, biochar-based or biochar-blended fertilizers, including biochar co-composted organic fertilizer, biochar urea, and biochar compound fertilizers offer opportunities to apply biochar to fields through conventional farm fertilization methods [14]. The biochar fertilizer effect has been increasingly attributed to the habitat modulation of the biochar material [4,10,11,15]. However, nutrient adsorption by biochar may only have a limited effect [16] given its unique performance in enhancing plant growth and nutrient use efficiency [17]. As early as 2015, Lehmann et al. [18] pointed out that the soil–plant interplay could be the core of biochar’s role in agriculture when using biochar or biochar-based fertilizers (Figure 1). However, limited progress has been made in understanding and characterizing such interplays and hidden mechanisms.

With the fast-growing global demand for bioenergy and net-zero food production [19], the use of biochar in agriculture is expected to expand worldwide. Hence, it is crucial to understand the interplay between biochar, soil, and plants. Here, the effect of biochar on microbial activity that controls plant growth, plant health, and, thus, food quality, is preponderant. In our Special Issue on “Biochar-Based Fertilizers in Agriculture: Soil–Plant Interactions and Functions”, ten papers were included to address the agronomic effects (productivity, quality, and growth performance) and soil–microbe–plant interactions following biochar-based amendments and fertilizers. Herein, biochar’s ability to improve soil conditions for plant growth is reported for ornamental plants [20], rice seedlings [21], wheat [15,22], vegetables [9], radish [23], tobacco [24], and peanuts [12]. The published studies also report biochar’s role in remediating organic and inorganic soil pollutants in vegetable [25], maize [26], and rice [27] cultivation. These studies highlight the role of biochar or biochar fertilizers in improving plant tolerance to pollution stress [12,25,28] and also in improving resilience to drought and salinity stresses [11,28,29]. The lessons learned from these studies include, but are not limited to, the following:

(1) In addition to biochar nano-particles [6,19,22,24], biomolecules from pyrolytic liquids [30], often called wood vinegar, may contribute to plant growth promotion [20];
(2) Plant responses such as growth and metabolism (the latter positively affecting food quality) can be coupled or uncoupled, depending on biochar properties, as well as soil and plant conditions [12,20,21,22,23];
(3) Biochar acts via its pore volume and surface area, as a carrier of moisture and nutrients, and enhancer of microbial activity, affecting plant growth and activity directly [15,21,23,24,25];
(4) Biochar-induced plant resilience could be attributed to a general improvement of soil physical, chemical, and biological functions following its application [12,23,27].

Overall, the papers in the present Special Issue highlight biochar’s potential to enhance plant growth and resilience against adverse impacts resulting from environmental pollution and climate change. It is increasingly important that plant tolerance and resilience to adverse stress, such as drought and pollution, increasingly contribute to sustainable plant production under global climate change [15,31]. Unfortunately, the published studies also highlight uncertainty about the mechanisms by which to enhance or promote plant growth and resilience. Following its application, biochar may induce a direct or indirect improvement in the soil’s physical [13,16], chemical [11,16,20,22,25], and/or biological conditions [4,11,12,26,29]. However, the indirect impacts on plants remain unclear, as plant responses to biochar or biochar fertilizers require more in-depth investigations. Following the field application of biochar or biochar-based fertilizers, some systematic changes were observed in the rhizosphere (the soil–plant interaction zone) [11,12,13]. In this context, the interplay between the soil and plants following biochar or biochar fertilization application results in a very complex, dynamic, and multidimensional change in crop cultivation (Figure 2). These changes could be illustrated using the ancient Chinese theory of Yijing (also known as I Ching), considering how biochar could induce physical (structural), chemical, and biological changes in soil (the lower part in Figure 2) as well as systemic changes in the soil–plant system, which may alter various plant processes such as nutrition, growth, and resilience (the upper part in Figure 2). Soil changes and alterations in plant metabolism occur in parallel, while plant resilience can be considered the net result of plants’ responses to the altered soil–plant system following biochar or biochar fertilizer application (Figure 2). Biochar-induced soil changes have been most frequently addressed regarding physical change (e.g., bulk density, water retention), chemical change (e.g., pH), and biological change (e.g., microbial interaction) while the system change as a result of soil–plant interactions (the bottom right-hand corner) is still poorly understood. Meanwhile, plant responses to soil changes following biochar application have been unevenly addressed across areas of plant growth, nutrition, and protection, while changes in plant resilience (against pollution or adverse climate stress) due to the altered soil–plant system have not yet been thoroughly examined. Therefore, the publication of this Special Issue underscores the urgent need to investigate biochar-induced plant resilience in global plant production and food supply systems in a changing environment, amid increasing pollution and climate change.

The papers of this Special Issue provide evidence that biochar, particularly when combined with organic or inorganic fertilizers, improves soil fertility and plant health. It enhances food productivity of agro-ecosystems and plant resilience to adverse environmental impacts, such as pollutant toxicity, salinity, and drought stresses. Biochar, beyond its chemically and physically determined adsorption capacity [16], should be revalued as a multifunctional, carbon-rich, and persistent material with a porous structure and molecular biological activity. It acts as an eco-friendly plant modulator, both for growth promotion and metabolism. Through its role in bio-synthesis, bio-control, and/or bio-defense, biochar strengthens the amended ecosystems. While global societies are facing serious challenges due to climate change, land degradation, and environmental pollution, biochar can contribute to agricultural sustainability and assist plants in increasing resilience to adverse climate events. To enhance our understanding of biochar–soil–plant interactions and functions [18], more dedicated studies into molecular changes [32] and the signaling responses of soil–plant systems [4,29] following biochar application under field conditions are needed.

## Figures and Tables

**Figure 1:**
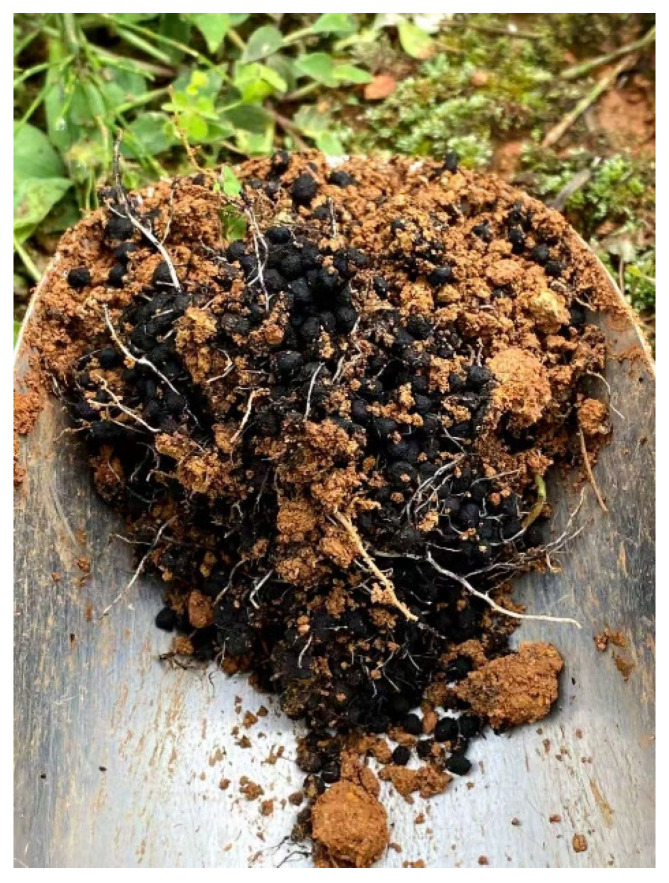
Tropical red soil with residual biochar pellets under a mango tree 6 months after application of a biochar compound fertilizer, showing the interplay between the soil mass, biochar fertilizer, and plant roots. Note that the plant’s fine roots are densely attached to the biochar pellets. Photo taken in 2022 by G. Pan.

**Figure 2:**
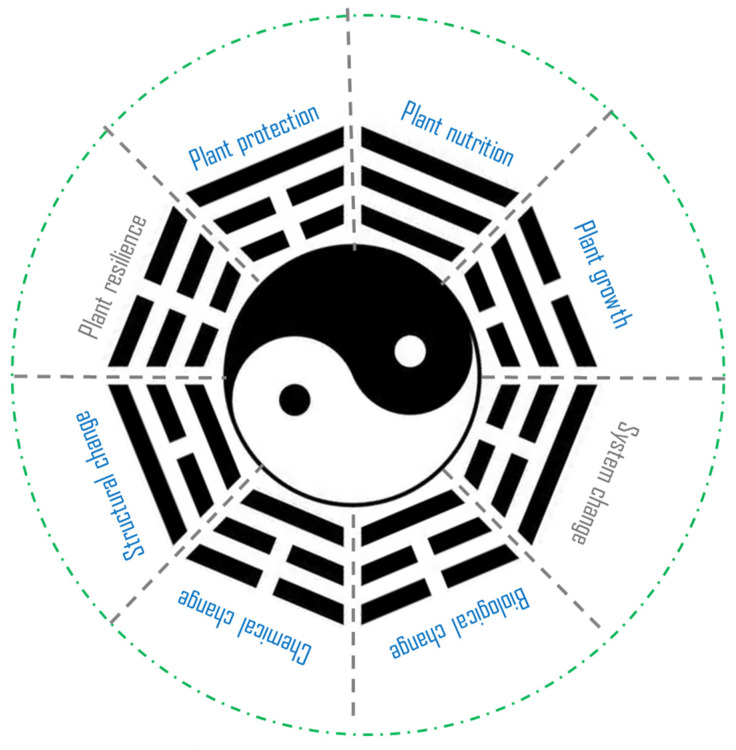
The complex, dynamic, and multidimensional change in the soil–plant system following biochar or biochar fertilizer application. Such a system is conceived as a Tai Ji system following the ancient Chinese theory of Yijing. Herein, soil performance is denoted by the white pole with the black organic matter as the core, while the plant performance is denoted by the black pole with the white root as the core. The width of a pole across the performance represents the size and clarity of the biochar effect. The unbroken black arcs represent clear positive influences, while the broken arcs represent negative or unclear influences.

## References

[1] FAO, IFAD, UNICEF, WFP, WHO (2023). The State of Food Security and Nutrition in the World 2023. Urbanization, Agrifood Systems Transformation and Healthy Diets Across the Rural–Urban Continuum.

[2] Keesstra S., Mol G., de Leeuw J., Okx J., Molenaar C., De Cleen M., Visser S. (2018). Soil-related sustainable development goals: Four concepts to make land degradation neutrality and restoration work. Land.

[3] Zhang J., Li C. (2023). Biochar for Sustainable Farming and Recultivation. Agronomy.

[4] Mehari Z.H., Elad Y., Rav-David D., Graber E.R., Harel Y.M. (2015). Induced systemic resistance in tomato (*Solanum lycopersicum*) against Botrytis cinerea by biochar amendment involves jasmonic acid signalling. Plant Soil.

[5] Nguyen T.-B., Sherpa K., Bui X.-T., Nguyen V.-T., Vo T.-D., Ho H.-T., Chen C.-W., Dong C.-D. (2023). Biochar for soil remediation: A comprehensive review of current research on pollutant removal. Environ. Pollut..

[6] Pan G., Bian R., Cheng K. (2017). From biowaste treatment to novel bio-material manufacturing: Biomaterial science and technology based on biomass pyrolysis. Sci. Technol. Rev..

[7] Bolan N., Hoang S.A., Beiyuan J., Gupta S., Hou D., Karakoti A., Joseph S., Jung S., Kim K.-H., Kirkham M. (2021). Multifunctional applications of biochar beyond carbon storage. Int. Mater. Rev..

[8] Cheema A.I., Amina, Ullah H., Munir M.A.M., Rehman A., Sarma H., Pikoń K., Yousaf B. (2024). Unraveling the mechanisms of free radicals-based transformation and accumulation of potentially toxic metal(loid)s in biochar- and compost-amended soil-plant systems. J. Clean. Prod..

[9] Zhuang Q.-L., Yuan H.-Y., Sun M., Deng H.-G., Zama E.F., Tao B.-X., Zhang B.-H. (2025). Biochar-mediated remediation of low-density polyethylene microplastic-polluted soil-plant systems: Role of phosphorus and protist community responses. J. Hazard. Mater..

[10] Bolan S., Sharma S., Mukherjee S., Kumar M., Rao C.S., Nataraj K., Singh G., Vinu A., Bhowmik A., Sharma H. (2023). Biochar modulating soil biological health: A review. Sci. Total Environ..

[11] Liu C., Xia R., Tang M., Liu X., Bian R., Yang L., Zheng J., Cheng K., Zhang X., Drosos M. (2022). More microbial manipulation and plant defense than soil fertility for biochar in food production: A field experiment of replanted ginseng with different biochars. Front. Microbiol..

[12] Liu C., Shang S., Wang C., Tian J., Zhang L., Liu X., Bian R., He Q., Zhang F., Chen L. (2025). Biochar amendment increases peanut production through improvement of the extracellular enzyme activities and microbial community composition in replanted field. Plants.

[13] Lu H., Bian R., Xin X., Cheng K., Liu X., Liu Y., Wang P., Li Z., Zheng J., Zhang X. (2020). Legacy of soil health improvement with carbon increase following one time amendment of biochar in a paddy soil—A rice farm trial. Geoderma.

[14] Joseph S., Graber E., Chia C., Munroe P., Donne S., Thomas T., Nielsen S., Marjo C., Rutlidge H., Pan G. (2013). Shifting paradigms: Development of high-efficiency biochar fertilizers based on nano-structures and soluble components. Carbon Manag..

[15] Noureen S., Iqbal A., Muqeet H.A. (2024). Potential of drought tolerant rhizobacteria amended with biochar on growth promotion in wheat. Plants.

[16] Rasse D.P., Weldon S., Joner E.J., Joseph S., Kammann C.I., Liu X., O’tOole A., Pan G., Kocatürk-Schumacher N.P. (2022). Enhancing plant N uptake with biochar-based fertilizers: Limitation of sorption and prospects. Plant Soil.

[17] Zheng J., Han J., Liu Z., Xia W., Zhang X., Li L., Liu X., Bian R., Cheng K., Zheng J. (2017). Biochar compound fertilizer increases nitrogen productivity and economic benefits but decreases carbon emission of maize production. Agric. Ecosyst. Environ..

[18] Lehmann J., Kuzyakov Y., Pan G., Ok Y.S. (2015). Biochars and the plant-soil interface. Plant Soil.

[19] Bilotto F., Christie-Whitehead K.M., Barnes N., Harrison M.T. (2024). Operationalising net-zero with biochar: Black gold or red herring?. Trends Food Sci. Technol..

[20] Iacomino G., Cozzolino A., Idbella M., Amoroso G., Bertoli T., Bonanomi G., Motti R. (2023). Potential of biochar as a peat substitute in growth media for *Lavandula angustifolia*, *Salvia rosmarinus* and *Fragaria ananassa*. Plants.

[21] Zhang K., Han X., Fu Y., Zhou Y., Khan Z., Bi J., Hu L., Luo L. (2023). Biochar coating as a cost-effective delivery approach to promoting seed quality, rice germination, and seedling establishment. Plants.

[22] Shani M.Y., Ahmad S., Ashraf M.Y., Nawaz M., Arshad I., Anjum A., De Mastro F., Cocozza C., Khan Z., Gul N. (2024). Nano-biochar suspension mediated alterations in growth, physio-biochemical activities and nutrient content in wheat (*Triticum aestivum* L.) at the vegetative stage. Plants.

[23] Mon W.W., Ueno H. (2024). Short-term effect of the combined application of rice husk biochar and organic and inorganic fertilizers on radish growth and nitrogen use efficiency. Plants.

[24] Yang L., Li S., Ahmed W., Jiang T., Mei F., Hu X., Liu W., Abbas F.M., Xue R., Peng X. (2024). Exploring the relationship between biochar pore structure and microbial community composition in promoting tobacco growth. Plants.

[25] Huang L., Chen W., Wei L., Li X., Huang Y., Huang Q., Liu C., Liu Z. (2024). Biochar blended with alkaline mineral can better inhibit lead and cadmium uptake and promote the growth of vegetables. Plants.

[26] Ma W., Luo P., Ahmed S., Hayat H.S., Anjum S.A., Nian L., Wu J., Wei Y., Ba W., Haider F.U. (2024). Synergistic effect of biochar, phosphate fertilizer, and phosphorous solubilizing bacteria for mitigating cadmium (Cd)stress and improving maize growth in Cd-contaminated soil. Plants.

[27] Yang Y., Liu L., Xiong H., Wang T., Yang J., Wang W., Al-Khalaf A.A., Wang Z., Ahmed W. (2025). Biochar and trehalose co-application: A sustainable strategy for alleviating lead toxicity in rice. Plants.

[28] Tripti, Kumar A., Maleva M., Borisova G., Rajkumar M. (2023). Amaranthus biochar-based microbial cell composites for alleviation of drought and cadmium stress: A novel bioremediation approach. Plants.

[29] Murtaza G., Ahmed Z., Usman M., Zaman Q.U., Deng G., Chen S., Alwahibi M.S., Rizwana H., Iqbal J., Ahmad S. (2025). Protective effects of multiple-chemical engineered biochar on hormonal signalling, antioxidant pathways and secondary metabolites in Lavender exposed to chromium and fluoride toxicity. J. Crop. Health.

[30] Jing J., Chen S., Wang Y., Zhao Z. (2024). Organic and inorganic composition of three typical pyrolitic liquids and their effect on capsicium growth with foliar fertilization. Chin. J. Agric. Resour. Environ..

[31] Zhao Z., Liu C., Yan M., Pan G. (2023). Understanding and enhancing soil conservation of water and life. Soil Sci. Environ..

[32] Azeem M., Wang J., Kubwimana J.J., Kazmi S.S., Khan Z.H., He K., Han R. (2025). Biochar-derived dissolved organic matter (BDOM) shifts fungal community composition: BDOM-soil DOM interaction. Appl. Soil Ecol..

